# Chronic inflammation is associated with neural responses to faces in bangladeshi children

**DOI:** 10.1016/j.neuroimage.2019.116110

**Published:** 2019-11-15

**Authors:** Wanze Xie, Swapna Kumar, Shahria H. Kakon, Rashidul Haque, William A. Petri, Charles A. Nelson

**Affiliations:** a Boston Children’s Hospital, Boston, MA, USA; b Harvard Medical School, Boston, MA, USA; c ICDDR, B, Dhaka, Bangladesh; d University of Virginia, Infectious Diseases & International Health, Charlottesville, VA, USA; e Harvard Graduate School of Education, Cambridge, MA, USA

**Keywords:** Early-life inflammation, ERPs, Cognitive development, Low-income countries

## Abstract

**Background:**

Early exposure to inflammation in childhood is increasingly recognized as one of the major factors that hinder millions of children worldwide from meeting their full developmental potential. The current study examined the association between systemic inflammation and children’s neural responses to facial stimuli and explored if this activity mediated the relation between inflammation and cognitive outcomes.

**Method:**

Two separate cohorts of children living in an urban slum in Dhaka, Bangladesh who are at high-risk for sustained inflammation were recruited in this study. The concentration of C-reactive protein (CRP) in blood samples served as our index of inflammation. Blood samples were collected once at 18 weeks for the younger (infant) cohort (N = 125) and at 6, 18, 40, 53, and 104 weeks for the older (toddler) cohort (N = 120). Event-related potentials (ERPs) were also recorded separately for the two cohorts: at 6 months for the younger cohort (N = 48) and at 36 months for the older cohort (N = 93), using a face-oddball paradigm in which standard and oddball faces were presented. Cognitive outcomes were evaluated with Mullen Scales of Early Learning (MSEL) at 27 months for the younger cohort (N = 98) and with Wechsler Preschool and Primary Scale of Intelligence (WPPSI) at 48 months for the older cohort (N = 124).

**Results:**

For the older toddler cohort, the P400 and Nc amplitude differences between the two types of stimuli were found to be associated with the frequency of elevated CRP such that more episodes of elevated CRP corresponded to smaller P400 and Nc differences between the two conditions. In addition, the P400 and Nc differences were both found to mediate the relation between inflammation and performance IQ scores. For the younger infant cohort, the participants showed differentiated N290 response to the two types of stimuli, but no association between the ERP response and CRP concentration was found.

**Conclusions:**

These findings suggest that chronic systemic inflammation has a long-term impact on children’s brain functioning and cognitive development. The neural circuitries associated with social attention and recognition memory of faces may be potential pathways by which inflammation exerts its effect on cognitive development.

## Highlights

• Chronic inflammation is assoicated with ERP responses to faces in Bangladeshi children.
• Children more frequently having elevated CRP level show smaller ERP difference between familiar and novel faces.
• The ERP difference is associated with future IQ.
• The ERP activation mediates the relation between inflammation and future IQ.

## 1 Introduction

Inflammation is one of the key mechanisms through which infectious diseases impact neurodevelopment. Systemic inflammation is commonly accompanied by infections as the result of the release of pro-inflammatory cytokines and proteins (Sankowski et al., 2015). The impact of long-lasting or chronic systemic inflammation on child cognitive development is increasingly recognized as one of the major factors that hinder millions of children worldwide from meeting their full developmental potential (Grantham-McGregor et al., 2007; Jiang et al., 2018), especially for children living in low-income countries (Walker et al., 2007). However, the neural mechanism by which this early adverse experience becomes “neurobiologically embedded” to produce alterations in children’s brain functioning are not known (Berens et al., 2017; Jensen et al., 2017). This could partially be due to the difficulty in collecting blood samples of children at high risk for inflammation, along with direct measurements of their brain development. The current study aimed to fill this gap by collecting blood samples from children living in an urban slum in Dhaka, Bangladesh who are at high-risk for sustained inflammation. Longitudinal blood samples were collected for a cohort of children in their first two years of life and one blood sample was collected for a different cohort of children at 18 weeks. We recorded event-related potentials (ERPs) in response to facial stimuli and examined the association between the measures of inflammation and brain’s electrical activity and overall cognitive function later in life.

There are different pathways by which systemic inflammation can communicate with the brain. Systemic inflammation is characterized by increased inflammatory cytokines (e.g., interleukin-1β (IL-1β), IL-6), proteins (e.g., C-reactive protein (CRP)) and other immunologically active peptides generated at the site of infection (Konsman et al., 2002). These pro-inflammatory mediators, in turn, can induce neuronal dysfunction by activating microglia in the brain, interfering with neuronal homeostasis and disrupting the neuronal milieu (Bilbo, 2010; Bilbo and Schwarz, 2009; Sankowski et al., 2015). Systemic inflammation can also lead to alterations of the permeability of the BBB (Varatharaj and Galea, 2017). The effects of inflammation observed at the molecular level may in turn contribute to changes in brain structure. An inverse association has been observed between plasma IL-6 level and gray matter (GM) volume in hippocampus and prefrontal cortex in adults (Marsland et al., 2008). In adults, increased CRP concentration in plasma is found to be associated with decreased white matter (WM) tract integrity, i.e., higher cost of directional diffusion of water along the nerve fibers (Gianaros et al., 2013), which is likely to be due to the delayed myelination that occurs under inflammatory conditions (Correale et al., 2019; Friese et al., 2014). Finally, systemic and intestinal inflammation are commonly associated with environmental enteropathy (EE), a subclinical condition due to constant fecal-oral contamination, in children living in low-income countries (Korpe and Petri, 2012). Early-life inflammation associated with EE has been shown to reduce the absorption of nutrients in the gut and consequently give rise to the development of malnutrition (Haque et al., 2014; Naylor et al., 2015; Scaldaferri et al., 2017), which in turn may compromise brain health and cognitive development (Jiang et al., 2018; Prado and Dewey, 2014).

Connections between early-life inflammation and neurodevelopmental impairments in children are increasingly inferred from behavioral studies (Dickson et al., 2000; Jiang et al., 2017). For instance, elevated concentration of inflammatory proteins shortly after childbirth was associated with poorer cognitive performance at two years in a cohort of preterm children born in North America (O’Shea et al., 2012). What remains unclear are the neural mechanisms underlying the effects of early-life inflammation on child behavior, particularly among children living in low-resource settings where enteric disease and malnutrition are common. Although it has recently been shown that maternal inflammation is associated with disrupted functional connectivity in memory- and attention-related brain networks in infants (Rudolph et al., 2018; Spann et al., 2018), to the best of our knowledge, no study has examined the association between postnatal inflammation and children’s brain functioning with neuroimaging tools.

Face perception and social attention are fundamental skills for children to master in order to learn and interact effectively in a social world. The investigation of how early-life inflammation may impact the neural correlates of these cognitive processes is critical for understanding the effect of early-life inflammation on child cognitive development. The face-oddball paradigm in which frequent (i.e., familiar, 70%) and oddball (i.e., novel, 30%) faces are presented to participants has been widely employed to examine the neural correlates of social attention and recognition memory of faces in children (Reynolds and Richards, 2005; Richards, 2003; Thomas and Nelson, 1996). In particular, three ERP components – the N290, P400 and negative-central (Nc) – can be elicited and detected using this paradigm from 3 to 6 months of age. The N290 component is regarded as the precursor of the adult N170 face-sensitive component, and its cortical sources are localized to the fusiform face and occipital face areas in children (Guy et al., 2016; Halit et al., 2004; Xie et al., 2018b). The P400 and Nc components have been regarded as the neural markers of sustained attention and attention allocation (the posterior orienting system) in children, as their amplitudes were observed to be larger in response to salient and novel stimuli, such as infrequently compared to frequently presented stimuli or faces compared to objects (de Haan and Nelson, 1997; Reynolds and Richards, 2005; Xie and Richards, 2016). A differential response in these ERP components between the two experimental conditions indicates the detection or discrimination of the infrequent from the frequent faces by the brain and reflects some aspect of memory updating and the efficiency of stimulus processing (de Haan and Nelson, 1997; Reynolds and Richards, 2017; Thomas and Nelson, 1996).

The objective of the current study was to investigate the effect of early-life inflammation on brain functioning and to explore how brain functioning mediates the relation between inflammation and cognitive outcomes. The concentration of CRP in peripheral blood samples was measured as the biomarker of systemic inflammation at 18 weeks for the infant cohort and at 5 consecutive times during the first two years of life for the toddler cohort. Children’s brain functioning was assessed by measuring differences in the brain’s electrical activity (ERPs) in the face-oddball task. For the infant cohort, we evaluated their cognitive outcome at 27 months of age using Mullen Scales of Early Learning (MSEL) (Mullen, 1995). For the toddler cohort, we assessed their intelligence scores at 48 months with Wechsler Preschool and Primary Scale of Intelligence (WPPSI-III) (Wechsler, 1991). We hypothesized that higher CRP concentration (infant cohort) and more episodes of elevated CRP (toddler cohort) would both be prospectively associated with smaller ERP differences between familiar and novel faces measured later in life. We further hypothesized that the ERP responses would be associated with cognitiv outcomes and mediate the relation between systemic inflammation and cognitive outcomes. Since inflammation caused by enteric diseases has been shown to influence cognitive development though malnutrition (Haque et al., 2014; Jiang et al., 2018), we included the height-for-age Z-scores (HAZ), a widely used indicator of malnutrition, as a covariate and an additional mediator in the analyses.

## 2 Method

### 2.1 Participants

The infant cohort consisted of 130 (56M/74F) participants who were recruited from the “Cryptosporidium Burden” (CRYPTO) study and had their blood sample collected at 18-weeks of age, height measured at 4.5 (M = 4.31, SD = 0.01) and 6 months, ERP tested at 6 months (M = 6.09, SD = 0.13) and cognitive assessment at 27 months (M = 26.84, SD = 2.41). The toddler cohort consisted of 130 (72M/58F) participants who were recruited from the “Performance of Rotavirus and Oral Polio Vaccines in Developing Countries” (PROVIDE) study had their blood samples collected at 6, 18, 40, 53, and 104 weeks, height measured at 21 (M = 21.33, SD = 0.07), 30 (M = 30.80, SD = 0.26) and 36 (M = 36.88, SD = 0.19) months ERP tested at 36 months (M = 36.88, SD = 0.19) and cognitive assessment at 48 months (M = 48.46, SD = 0.20). Participants’ HAZ scores were calculated based on World Health Organization (WHO) standards for each time point and then averaged across the time points. All infants and toddlers were born ≥ 34 gestational weeks. These participants had no known history of neurological abnormalities, traumatic brain injury, genetic disorders, or visual or auditory impairments.

The two cohorts of participants were recruited from an impoverished neighborhood (urban slum) in Dhaka, Bangladesh. Children living there are often exposed to air and water pollution, significant poverty and unsanitary living conditions. These environmental hazards have been associated with various kinds of health issues including growth faltering, enteric diseases and inflammation-related brain injury (Jiang et al., 2014, 2017). The children involved in the CRYPTO and PROVIDE studies received all standard vaccines included in the national Bangladesh Expanded Program on Immunization (EPI); they also received free medical care for the duration of the study, which included treatment for diarrheal, respiratory and febrile illnesses that could cause acute or chronic inflammation (Kirkpatrick et al., 2015). The average monthly household income for the two cohorts was the equivalent of $187 (SD = 119) and $154 (SD = 107), respectively. We further defined a latent factor to index the socioeconomic status (SES) of each family, which was calculated based on multiple correlated indicators including income-to-needs quartiles, house construction materials, and family assets. The SES was comparable between the infant (M = 10.01, SD = 8.28) and toddler (M = 9.90, SD = 7.45) cohorts.

Ethical approval for the study was obtained from research review and ethics review committees at the International Centre for Diarrheal Disease Research, Bangladesh and Institutional Review Boards at Boston Children’s Hospital and were in accordance with local guidelines and regulations. We have collected written consent forms from all families who participated in the study.

### 2.2 Stimuli and task procedure

**Fig. 1:**
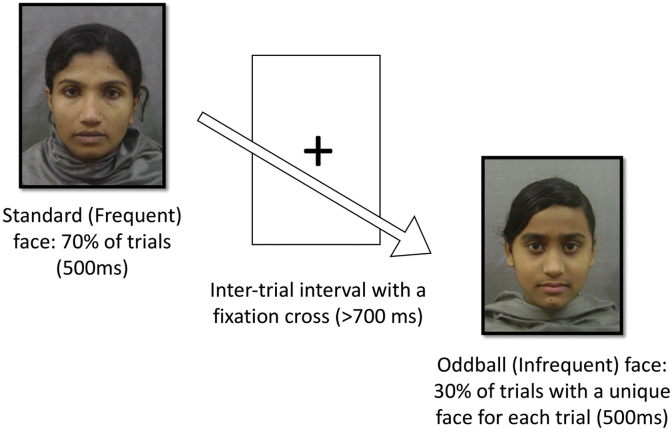
The face-oddball paradigm used in the current study.

Continuous EEG was recorded while the participants were seated on a caregiver’s lap approximately 65 cm from the presentation screen. The face-oddball task is illustrated in Fig. 1. The ERPs were derived from the continuous EEG recordings by averaging the segmented EEG data across all trials (events) for each condition. The stimuli used in this task included 46 images of local Bangladeshi female faces with a neutral expression. Image presentation was controlled using E-Prime 2.0 (Psychological Software Products, Harrisburg, PA). Stimuli were presented on a gray background subtended 14.3° × 12.2° of visual angle. Each stimulus was presented for 500 ms, followed by a fixation cross. The minimum inter-stimulus interval was 700 ms. For 6-month-old infants, the cross remained on the screen until the experimenter presented the next trial depending on the infants’ looking behavior. For 36-month-old toddlers, the presentations advanced automatically. Two of the 46 faces were used as the standard faces, and one of them was randomly selected to be the standard stimulus in the experiment. The remaining images were used as the oddball stimuli. The oddball, i.e., infrequent faces were presented for 30% of the trials and each trial consisted of a different face, while the standard, i.e., frequent face was presented for the other 70% of trials. The presentation continued until the maximum number of trials (N = 150) had been reached, or until the child’s attention could no longer be maintained. A second experimenter was seated next to the child for the duration of testing to assist in directing the infant’s attention to the presentation screen when necessary. Participants’ looking behavior was monitored and recorded using a video camera.

### 2.3 EEG recording, processing, and analysis

EEG was recorded from a 128-channel HydroCel Geodesic Sensor Net (HGSN) that was connected to a NetAmps 300 amplifier (Electrical Geodesic Inc., Eugene, OR). Channel impedances were kept at or below 100 kΩ and signals were sampled at 500 Hz. EEG recordings were filtered with a 0.3–30 Hz finite impulse response (FIR) band-pass filter and segmented from 100 ms before stimulus onset as the baseline to 900 ms following stimulus onset. The segmented epochs were inspected for artifacts automatically in ERPLAB (Lopez-Calderon and Luck, 2014) using both absolute (EEG amplitude < −100 or >100 μV) and stepwise (ΔEEG > 100 μV in a moving window with 100 ms as the size of the window and 50 ms as the length for each step) algorithms. Visual inspection of the epochs was also performed to mark those containing eye blinks, eye movements, no looking behavior, or drift in the signal. Bad epochs identified by either procedure were excluded from further analysis. An entire epoch was excluded if more than 18 electrodes (15%) overall had been rejected (Luyster et al., 2014; Xie et al., 2018b). Of the remaining trials, individual electrodes containing artifacts were replaced using a linear interpolation with the five closest good electrodes. The final data were re-referenced using the average reference. An equal number of “artifact-free” trials were then selected from the two conditions by randomly rejecting trials from the condition with more trials to rule out the effect of number of trials on the ERP results. Each participant was required to contribute at least 10 artifact-free trials per condition to be included in the final analysis. The average number of trials per condition was not different between the two different age groups, 6 mos: M = 20.69, SD = 7.59; 36 mos: M = 22.56, SD = 8.03.

Forty-eight of the original 130 infants had usable ERP data. Among the 82 excluded infants, 22 did not participate in the EEG portion of the study, one infant’s data were not collected correctly due to an experimenter error, and 59 did not have enough ERP trials during data collection or after artifact rejection for at least one condition. There were 93 out of the original 130 toddlers having usable ERP data. One participant did not participate in the EEG section, 3 participants’ data were rejected because of experimenter error or equipment failure, and 33 participants had not enough ERP trials for at least one condition.

The electrodes were combined into different electrode clusters covering the commonly studied scalp regions in previous examination of the N170/N290, P400, and Nc components in child face processing (Guy et al., 2016; Leppänen et al., 2007; Rigato et al., 2010; Xie et al., 2018b). The “Fronto-Central” cluster was used for the Nc analysis, the average of the “Occipito-Temporal_L″ and “Occipito-Temporal_R″ clusters were used for the N290 analysis, and the “Occipital-Inion” cluster was used for the P400 analysis (Supplemental Fig. 1). The N290 peak amplitude was measured in the time window of 190–290 ms and was corrected for the pre-N290 positive peak for the 6 months cohort by measuring the peak-to-peak amplitude (Guy et al., 2016; Kuefner et al., 2010; Xie et al., 2018b; Xie and Richards, 2017). The mean amplitude was computed for the P400 and the Nc components in the time window of 350–650 ms to better quantify their prolonged activation (Xie et al., 2018b).

### 2.4 Measurement of inflammation

The concentration of CRP in peripheral blood samples was measured as the index of inflammation. The blood sample for CRP assessment was collected at 18 weeks for the infant cohort and at 6, 18, 40, 53, and 104 weeks for the older toddler cohort. We evaluated the chronic (persistent) inflammation burden for the 36-month-old cohort by calculating the cumulative number of times the child experienced elevated CRP level. CRP elevation was defined as the child’s CRP concentration (mg/L) being at the top 50% in the group at the measurement time (Jiang et al., 2017; Naylor et al., 2015). As a result, the cumulative elevated CRP index varied between 0 and 5 since the CRP level was measured for 5 times before the EEG assessment. There were 120 out of 130 children in the older cohort that had usable CRP data for all 5 times. The remaining 10 children missed one to two CRP concentrations due to unusable blood samples randomly at different times. Multiple imputation with the algorithm of fully conditional specification was conducted in SPSS (version 25) for 100 times to impute the missing values using all available CRP data. The frequency of CRP elevation for the 10 children was calculated for each imputation and then the pooled mean frequency value (over 100 times) was estimated.

For the 6-month-old cohort, the CRP concentration at 18 weeks was log transformed to create a continuous and normally distributed measure for 125 infants who had usable blood samples. We were not able to evaluate the level of chronic inflammation for this younger cohort because of the only CRP measurement before the ERP test.

### 2.5 Cognitive assessment

The cognitive outcome of the infant cohort was assessed with Mullen Scales of Early Learning (MSEL) (Mullen, 1995) at 27 months for 98 out of the original 130 infants. The missing data were due to not attending the follow-up visit at 27 months (N = 14) or incomplete MSEL test (N = 18). The scores for four subscales (fine motor, visual reception, receptive language, and expressive language) were standardized and used to calculate a composite score. The score for visual reception subscale was used for further analysis because it was hypothesized to be more relevant to the cognitive processes, i.e., visual attention and face recognition, tested by the current ERP paradigm. The MSEL composite score was also used as an index of global cognitive development in further analysis.

Given a large portion of infants ended up with no usable ERP or MSEL data, CRP concentration was compared between infants with and without usable data to rule out the possibility that infants with higher CRP concentration were more likely to have low-quality ERP and behavioral data. No difference in CRP concentration was found between infants with and without usable ERP data, t = 0.027, 95%CI = [-0.536 0.557], *p* = .979. CRP concentration was slightly higher for infants with than without MSEL data, but the difference was not significant either, t = 1.68, 95%CI = [-0.091 1.122], *p* = .095.

The cognitive outcome of the toddler cohort was assessed with Wechesler Preschool and Primary Scalre of Intelligence (WPPSI-III) (Wechsler, 1991) at 48 months, as children older than 3 years of age tended to demonstrate a ceiling effect on MSEL. The WPPSI data were collected for 124 out of the original 130 children, and the missing data were due to incomplete WPPSI test. The performance IQ (PIQ) score was used for further analysis, as it is hypothesized to be more related to child non-verbal skills and visual attention that are more likely to be associated with the ERP components tested in the current study. The full-scale Intelligence Quotient (FSIQ) score, a reliable and representative measure of general intellectual functioning, was also calculated and used for further analysis. The MSEL and WPPSI were both culturally adapted and administered by local psychologists (Xie et al., 2018a).

### 2.6 Design for statistical analysis

Repeated-measures analyses of variance (ANOVAs) were performed to test whether the participants would show different ERP responses to frequent and infrequent faces on the group level. The ERP amplitudes for the N290, P400, and Nc components were analyzed as the dependent variables separately in three ANOVAs, which included age (2: 6 and 36 months) as a between-subjects independent variable and stimulus condition (2: frequent and infrequent) as a within-subjects independent variable. Greenhouse-Geisser corrections were applied when the assumption of sphericity was violated. When significant (*p* < .05) main or interaction effects emerged, post hoc comparisons were conducted with a Bonferroni correction.

Multiple linear regression was performed to test the associations between inflammation and ERP response (the difference between frequent and infrequent conditions for the three components), with the factor of HAZ being included as an independent variable (covariate). Regression was also performed to test the associations of ERP response and inflammation with cognitive outcomes (MSEL composite score and Visual Reception t-score for the infant cohort; FSIQ and PIQ scores for the toddler cohort). Repeated-measures ANOVAs and regression analyses were run in IBM SPSS Statistics (version 25, IBM Corp, Armonk, NY).

Longitudinal path analysis was finally conducted with Mplus (version 7.4) to test the hypothesized indirect pathways by which inflammation could affect children’s cognitive outcomes. One pathway is through the ERP response, i.e., to test whether ERP response mediates the relation between inflammation and cognitive outcomes. The other pathway is through HAZ and ERP reponse, i.e., to test whether inflammation is associated with malnutrition, which in turn may impact ERP response and cognitive outcomes. Only participants with ERP data were included in the analysis. Missing values for WPPSI scores (N = 5) for the toddler cohort were handled using full-information maximum likelihood (FIML) estimation with robust standard errors. Model fit was evaluated based on a non-significant *X*^2^ (p > .05), CFI > 0.95, SRMR < 0.08, and RMSEA < 0.06. Indirect effects were estimated using bootstrapping across 10,000 draws with bias-corrected confidence intervals.

## 3 Results

### 3.1 Decriptive analysis of CRP measures

For the infant cohort, the mean/median of the CRP concentration at 18 weeks were 4.379/1.335 mg/L (SD = 8.605). For the toddler cohort, the mean/median of the CRP concentration at 6, 18, 40, 53, and 104 weeks were 0.423/.097 (SD = 1.083), 2.727/.549 (SD = 6.393), 5.354/1.712 (SD = 10.240), 3.831/1.106 (SD = 7.903), and 6.213/1.593 (SD = 8.605) mg/L. The frequency of CRP elevation varies between 0 and 5: 0 (N = 8), 1 (N = 19), 2 (N = 35), 3 (N = 34), 4 (N = 28), and 5 (N = 6).

### 3.2 General ERP results

#### 3.2.1 N290 component

**Fig. 2:**
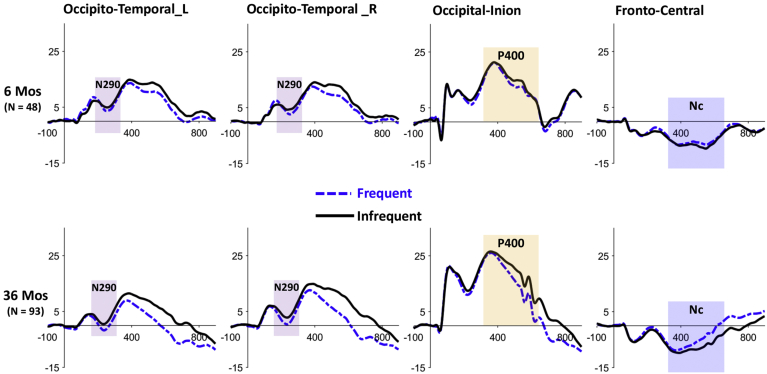
The grand-average ERP responses to the frequent (black & solid) and infrequent (blue & dash) faces in different electrode clusters, separately for the two age cohorts.

The repeated-measures ANOVA of the N290 component revealed a main effect of stimulus condition, F(1,139) = 17.694, *p* < .0001, η_p_^2^ = 0.113. The N290 amplitude for the frequent condition was greater (more negative) than that for the infrequent condition, M_diff_ = −1.868, 95% CI [-2.746 -.990]. There was no interaction between age and stimulus condition (Fig. 2).

#### 3.2.2 P400 component

The ANOVA of the P400 component revealed an interaction between age and stimulus condition, F(1,139) = 9.796, *p* = .002, η_p_^2^ = 0.066. Post-hoc comparisons with Bonferroni adjustment showed that the effect of stimulus condition was only significant for the 36 months old cohort, such that the P400 amplitude for the frequent condition was significantly smaller (less positive) than that for the infrequent condition, M_diff_ = −3.971, 95% CI [-5.052 -.2890] (Fig. 2). There was also a main effect of age, F(1,139) = 6.566, *p* = .011, η_p_^2^ = 0.045. The P400 amplitude was greater at 36 than 6 months, M_diff_ = 3.134, 95% CI [0.716 5.552].

#### 3.2.3 Nc component

The ANOVA of the Nc component also showed an interaction between age and stimulus condition, F(1,139) = 7.430, *p* = .007, η_p_^2^ = 0.051. Post-hoc comparisons showed that the Nc amplitude for the frequent condition was smaller (less negative) than that for the infrequent condition only for the 36 months old cohort, M_diff_ = 2.571, 95% CI [1.799 3.342] (Fig. 2). No effect of stimulus condition was found for the 6 months old cohort.

### 3.3 Association of inflammation with ERP responses

We further tested the association between inflammation and the ERP amplitude difference between the two conditions.

#### 3.3.1 Results for the infant cohort

Multiple linear regression analysis was performed to examine the associations of ERP responses with CRP concentration at 18 weeks and the average HAZ score between 4.5 and 6 months, separately for the N290, P400 and Nc components. The analyses revealed no effect of CRP concentration or HAZ on the ERP response for all three components.

#### 3.3.2 Results for the toddler cohort

The multiple linear regression analysis for the older cohort showed different results for three ERP components. The overall regression model for the N290 component was not significant F(2, 90) = 1.951, *p* = .148, R^2^ = 0.042, and neither frequency of CRP elevation (β = 0.209, *p* = .055) or average HAZ score from 21 to 36 months significantly predicted the N290 difference between the two conditions.

**Fig. 3:**
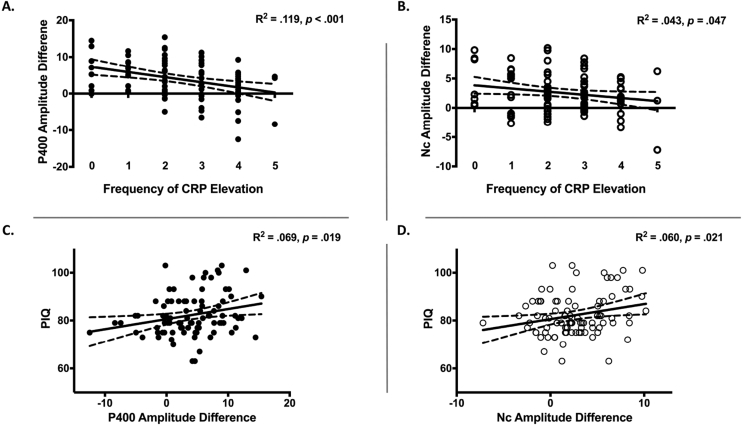
Linear regression lines that demonstrate the associations between the frequency of CRP elevation over the first two years of life and the P400 (A.) and Nc (B.) amplitude difference between the two conditions at 36 months, as well as the associations between the P400 (C.) and Nc (D.) amplitude difference and children’s performance IQ scores at 48 months.

**Fig. 4:**
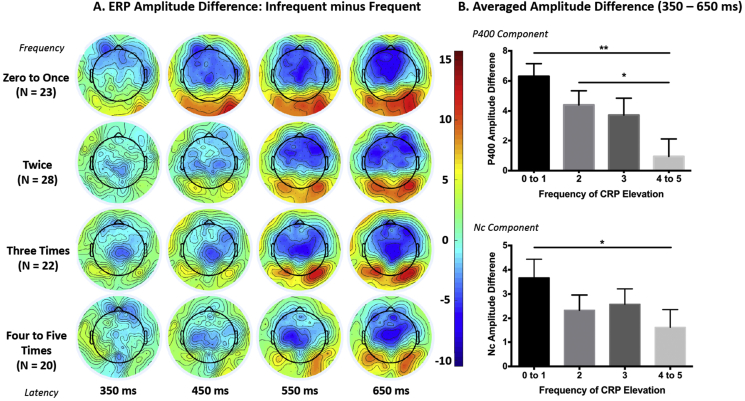
A. Topographical maps for the ERP difference between the two conditions, separately for different inflammation groups based on how many episodes they had elevated CRP level. The P400 component (positive) is most prominent in the occipital region, and the Nc component (negative) is most prominent in the central and frontal regions. The children with fewer episodes of elevated CRP (e.g., zero to once) showed greater P400 (more positive) and Nc (more negative) responses to the infrequent than frequent faces. B. Bar graphs showing the average amplitude difference across the entire P400/Nc time window as a function of how often the children had elevated CRP. Note: The Nc amplitude difference was calculated by subtracting the amplitude for the infrequent condition from that for the frequent condition, i.e., Frequent – Infrequent, and thus in both P400 and Nc graphs, a positive value mean greater response to infrequent faces. *p ​< ​.05, **p ​< ​.01. P-values for multiple comparisons were Bonferroni corrected.

The analysis for the P400 component revealed a significant regression equation, F(2, 90) = 6.089, *p* = .003, R^2^ = 0.119. The frequency of CRP elevation but not HAZ was significantly associated with the P400 difference between the two conditions, β = −0.337, *p* = .002, such that more episodes of elevated CRP corresponds to smaller P400 difference between the two conditions (Fig. 3A). In Fig. 4, the participants were further divided into four groups to obtain similar number of participants per group: children with zero or only once, twice, three times or more than four times of CRP elevation. It shows that the P400 difference for the children having the most frequent CRP elevation (i.e., >4 times) is close to zero and much smaller than that for the children with <3 times of CRP elevation (Fig. 4B).

The analysis for the Nc component revealed that the overall regression model is marginally significant, F(2, 90) = 3.075, *p* = .05, R^2^ = 0.064. Only the frequency of CRP elevation was a significant predictor of the Nc difference between the two conditions, β = −.250, *p* = .021, such that more episodes of elevated CRP corresponds to smaller Nc difference between the two conditions (Fig. 3, Fig. 4).

### 3.4 Associations of ERP and inflammation with future cognitive outcomes

Linear regression analyses were conducted to determine the assoication between inflammation and future cognitive outcomes. Our results showed that for the toddler cohort, the frequency of CRP elevation negatively predicted children’s PIQ [F(1,123) = 5.146, *p* = .025, β = −1.341, R^2^ = 0.201] and FSIQ [F(1,123) = 8.592, *p* = .004, β = −1.578, R^2^ = 0.257] at 48 months. No significiant association was found between the CRP concentration at 18 weeks and MSEL composite or visual reception scores at 27 months.

We further examined the association between the ERP responses and cognitive outcomes. The analysis for the toddler cohort showed that the P400 [F(1,88) = 5.652, *p* = .020, β = 0.420, R^2^ = 0.061] and Nc [F(1,88) = 5.511, *p* = .021, β = 0.626, R^2^ = 0.060] difference both positively predicted the PIQ but not FSIQ scores at 48 months, such that the greater the difference the higher the PIQ (Fig. 3C and D). There was no association found between the ERP response at 6 months and MSEL composite or visual reception scores at 27 months for the infant cohort.

### 3.5 Mediation model results

**Fig. 5:**
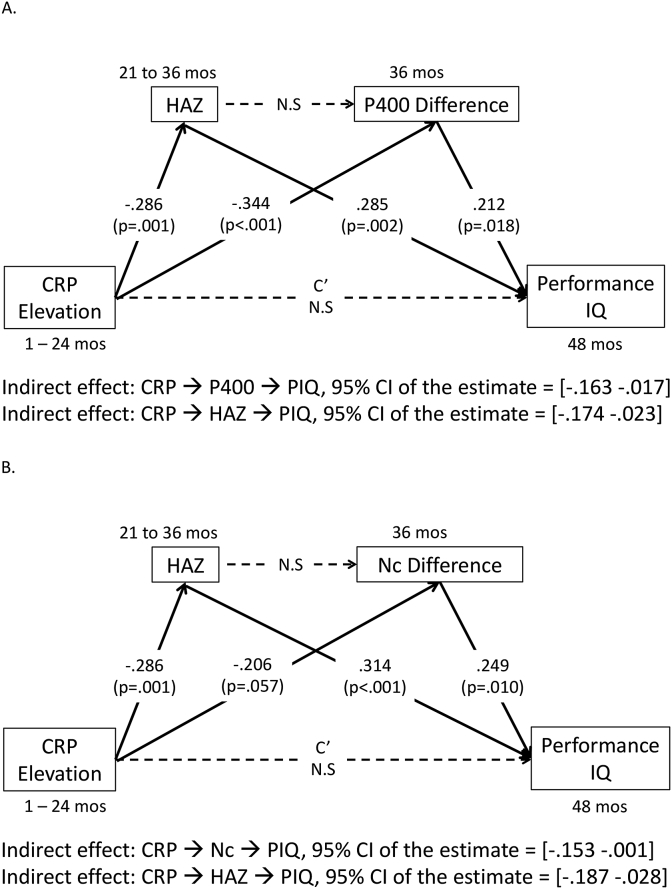
The two mediation models separately for the P400 (A.) and Nc (B.) responses.

Path analysis was run for the toddler not the infant cohort given the absence of associations between the factors for the latter. The indirect effect of inflammation on PIQ through the ERP responses was tested in the mediation model. The indirect effect of inflammation on PIQ through HAZ and ERP responses was not tested because there was no association between HAZ and ERP responses revealed by the regression analyses. The mediation model (Fig. 5) was run separately for the P400 and Nc components. The model fits are reported as following: the “P400 model”: χ^2^(1) = 0.074, p = .791; CFI = 1.00; SRMR = 0.008; RMSEA = 0.000; the “Nc model”: χ^2^(1) = 2.109, P = .146; CFI = 0.956; SRMR = 0.049; RMSEA = 0.109.

The path analysis for the P400 component revealed that the frequency of CRP elevation was negatively associated with the P400 difference, which in turn was positively associated with PIQ. The mediation effect of the P400 response on the relation between frequency of CRP elevation and PIQ was significant: 95% confidence interval (CI) of the estimate = [-0.163 -.017] (Fig. 5). The analysis for the Nc component revealed a similar mediation effect of the Nc response on the relation between frequency of CRP elevation and PIQ: 95% CI of the estimate = [-0.153 -.001]. In addition, both analyses revealed an indirect effect of HAZ on the relation between frequency of CRP elevation and PIQ (Fig. 5).

## 4 Discussion

The goal of the current study was to understand the long-term consequences of chronic systemic inflammation during infancy on the development of brain function in early childhood. We examined two groups of children living in an impoverished neighborhood in Dhaka, Bangladesh who are at high-risk for infectious diseases and chronic inflammation from early childhood. We measured an inflammatory marker – CRP concentration – along with ERP responses to familiar and novel (or “frequently and infrequently presented”) faces and cognitive scores at specific ages. As expected, the frequency of CRP elevation was negatively associated with the P400 and Nc differences between frequent and infrequent faces for the toddler cohort, such that more episodes of elevated CRP corresponded to smaller P400 and Nc differences. Children in the toddler cohort showing greater ERP evidence of discrimination between the two types of stimuli were found to have higher PIQ scores at 48 months. In addition, the ERP responses mediated the relation between the frequency of CRP elevation and PIQ scores; in other words, there was an indirect effect of chronic inflammation on PIQ through the ERP responses. In contrast to our hypothesis, the younger infant cohort whose CRP concentration was measured once at 18 weeks did not show an association between CRP concentration and ERP differences between conditions or a correlation between ERP responses and cognitive outcomes.

### 4.1 The association between inflammation and ERP responses

In the current study we provide the first empirical evidence for the association between chronic inflammation and children’s brain functioning measured with EEG-based tools – the ERP. The P400 and Nc components have been widely used as neural indices of stimulus processing and sustained attention in children (de Haan and Nelson, 1997; Richards, 2003; Xie and Richards, 2016), and their cortical sources have been localized to brain areas composing the default model and face networks (Guy et al., 2016; Xie et al., 2018b). Greater P400 and Nc responses to infrequent (novel) than frequent (familiar) faces are likely to reflect heightened cortical activation in these brain networks as a result of increased attention to novel faces (Thomas and Nelson, 1996; Xie and Richards, 2016), while smaller difference between the two conditions might suggest that the frequent face is processed to a less extent due to reduced neural efficiency, thereby the salience of the infrequent faces is reduced (Reynolds and Richards, 2017). The finding of a negative association between the frequency of CRP elevation and ERP differences (Fig. 3, Fig. 4) is consistent with our hypothesis. This finding suggests that the neural circuitry associated with face discrimination and orienting of attention are likely to be disrupted by early-life chronic systemic inflammation, especially for children with >4 times of elevated CRP in the first two years of life. This interpretation is in line with previous reports that maternal inflammation disrupts the functional connectivity in the attention-related salience (Spann et al., 2018) and memory (Rudolph et al., 2018) networks.

Children with <2 times of CRP elevation showed significant difference in the P400 and Nc components between the infrequent and frequent faces, and such difference reflects the ability to discriminate the infrequent from the frequent stimuli by the brain (de Haan and Nelson, 1997). This finding is consistent with what has been found in typically developing children in high-income countries (Reynolds and Richards, 2005). However, it is unlikely that the brain development of these Bangladeshi children and the function of their brain networks remain completely unaffected. It should be noted that elevations in CRP level were defined within the cohort by using the median CRP concentration as the threshold for elevated CRP per measurement (Jiang et al., 2017; Naylor et al., 2015). Further, the mean CRP levels measured at 40, 53, and 104 weeks were even higher than 3 mg/L, the normal threshold for minor CRP elevation, suggesting that systemic inflammation is likely to be a common issue for these children. Given the linear correlation found between the frequency of CRP elevation and ERP responses (Fig. 3A–B) and the fact that these children are living in an impoverished and unsanitary urban slum (Storrs, 2017), it is plausible that the brain functioning of the children with fewer times of CRP elevation is also affected by inflammation to some extent. Another line of future research may consider comparing these children at high-risk for inflammation to a local “upper-class” cohort with better living conditions.

We also hypothesized an association between frequency of elevated CRP and the N290 difference between the two conditions for the toddler cohort, however, this association only approached statistical significance level, β = 0.209, *p* = .055. The N290 component is regarded as the precursor of the adult N170 face-sensitive component (Halit et al., 2004), whose amplitude is greater in response to faces vs. non-faces and cortical sources are localized to the fusiform face and occipital face areas in children (Guy et al., 2016; Xie et al., 2018b). The marginally significant association might be due to insufficient statistical power. An alternative explanation is that the neural circuitries generating the N290 component developed earlier than the P400, Nc and the posterior orienting system, as a distinct N290 response to faces can already be detected in 3-month-old infants (Halit et al., 2004; Xie and Richards, 2016), while the modulation of the P400 and Nc amplitudes as a function of attention is not likely to be observed before 6-month-old (Reynolds and Richards, 2017; Xie and Richards, 2016) and continue to develop over the first two year of life (Goldman et al., 2004). As a result, the N290 component and its underlying brain circuitries could be more resilient to the impact of adverse experiences, and thus show a weaker association with the frequency of CRP elevation over the first two years of life.

The altered brain responses to infrequent vs. frequent facial stimuli might reflect fundamental changes in brain architecture and/or function as a result of chronic systemic inflammation. Elevated CRP has been shown to interfere with synaptic pruning (Stephan et al., 2012) and generation of WM tracks and axons (Gianaros et al., 2013), as well as drive the increase of the permeability of BBB over time (Elwood et al., 2017). These biological changes, especially reduced structural connectivity of WM tracks, may underlie the dysfunction of the neural circuitry associated with face processing and attention in children suffering from chronic inflammation. Because CRP elevation is often accompanied by an increase of the production of pro-inflammatory cytokines (Gabay and Kushner, 1999; Liu et al., 2010), the neuronal dysfunction and disrupted network connections caused by the release of cytokines (Bilbo and Schwarz, 2009; Sankowski et al., 2015) are very likely to co-exist in these children.

### 4.2 Potential mediators of the relation between inflammation and cognitive outcomes

The current study also adds to the literature by providing insight into a potential neural pathway by which early-life inflammation induces derailed cognitive development in childhood. The P400 and Nc differences between the two conditions measured at 36 months were found to be positively associated with children’s PIQ scores at 48 months, and these ERP responses were found to mediate the relation between early-life inflammation during infancy and intellectual development (Fig. 5). The PIQ domain on WIPPSI includes scales that assess children’s nonverbal reasoning, attention and visual-motor coordination skills. Our findings suggest that disrupted neural circuitries underlying face processing and sustained attention due to chronic inflammation early in life may give rise to delayed cognitive development in relevant domains.

We observed that children with multiple episodes of elevated CRP during infancy had lower intelligence (PIQ and FSIQ) scores at four years of age. This finding provides converging evidence for effects of early-life inflammation on neurocognitive development that have been reported previously (Dickson et al., 2000; Jiang et al., 2014, 2017; O’Shea et al., 2012). A recent study found a positive association between maternal inflammation and neurodevelopmental scores in infants, which was interpreted as reflecting an adaptive neurodevelopmental response to the presence of maternal inflammation in infants (Spann et al., 2018). This discrepancy suggests that the mechanisms underlying the effects of maternal and postnatal inflammation on infant brain development could be different, which in turn may lead to different cognitive outcomes. Our finding of the mediation effect of HAZ scores on the relation between inflammation and IQ scores supports the idea that malnutrition associated with systemic inflammation due to enteric diseases in children living in low-income countries could also affect neurocognitive development (Jiang et al., 2018). However, the lack of an association between HAZ scores and ERP responses is inconsistent with our hypothesis. It is possible that the effect of malnutrition on cognitive development has an independent pathway bypassing the brain networks associated with face processing and attention, or the impact of malnutrition takes time to accumulate given the IQ scores were measured a year later than the ERP responses.

### 4.3 The younger cohort and limitations

In contrast to our hypothesis, the infant cohort did not show an association of inflammation with their ERP responses or cognitive outcomes. It is possible that we were unable to detect an association between inflammation and brain activity at 6 months of age, as such an association may take time to manifest itself (i.e., with advancing age), or our ERP measure lacks the sensitivity to detect the derailed development of the neural circuits underlying social attention due to inflammation early in life. The non-significant association between inflammation and MSEL scores could be due to that tasks of MSEL are too demanding (e.g., the reliance on verbal prompts) for infants growing up in such low-resource settings who may be delayed in development in various cognitive domains. In addition, the only measurement of CRP level at 18 weeks for the infant cohort may not be able to account for the effect of chronic inflammation during the first few months of life. It is valuable to follow this younger cohort and measure their brain activity, cognitive performance and CRP concentration on additional time points to justify these explanations.

One limitation of the current study is limited control of other potential covariates in addition to stunting due to the (relatively) small sample size. These children are growing up in an impoverished neighbourhood who are likely to suffer from other adverse experiences as well, such as poverty or reduced caregiving quality (Nelson, 2017). Future research with larger sample size may consider exploring the associations between all these factors and teasing apart the contribution of each to the derailed brain development in children living in this kind of environment in low-income countries.

## 5 Conclusion

The current study demonstrates that chronic systemic inflammation during infancy has a long-term impact on children’s brain functioning and cognitive development. The neural circuitry associated with face processing and sustained attention are likely to be affected by early-life inflammation and be potential pathways by which inflammation derails cognitive development in relative domains. The measure of ERP with EEG recordings is shown to be a useful tool to study brain functioning in low-resource settings. Children growing up in these low-resource settings are at high-risk for inflammation-related health issues from early childhood, and thus immune-based therapies may be critical to prevent developmental delays.
