# Supplementary figures and images for: Chronic inflammation is associated with neural responses to faces in bangladeshi children
Source: Neuroimage. 2019 Nov 15;202:116110. doi: 10.1016/j.neuroimage.2019.116110 (PMC6853162; doi:10.1016/j.neuroimage.2019.116110)

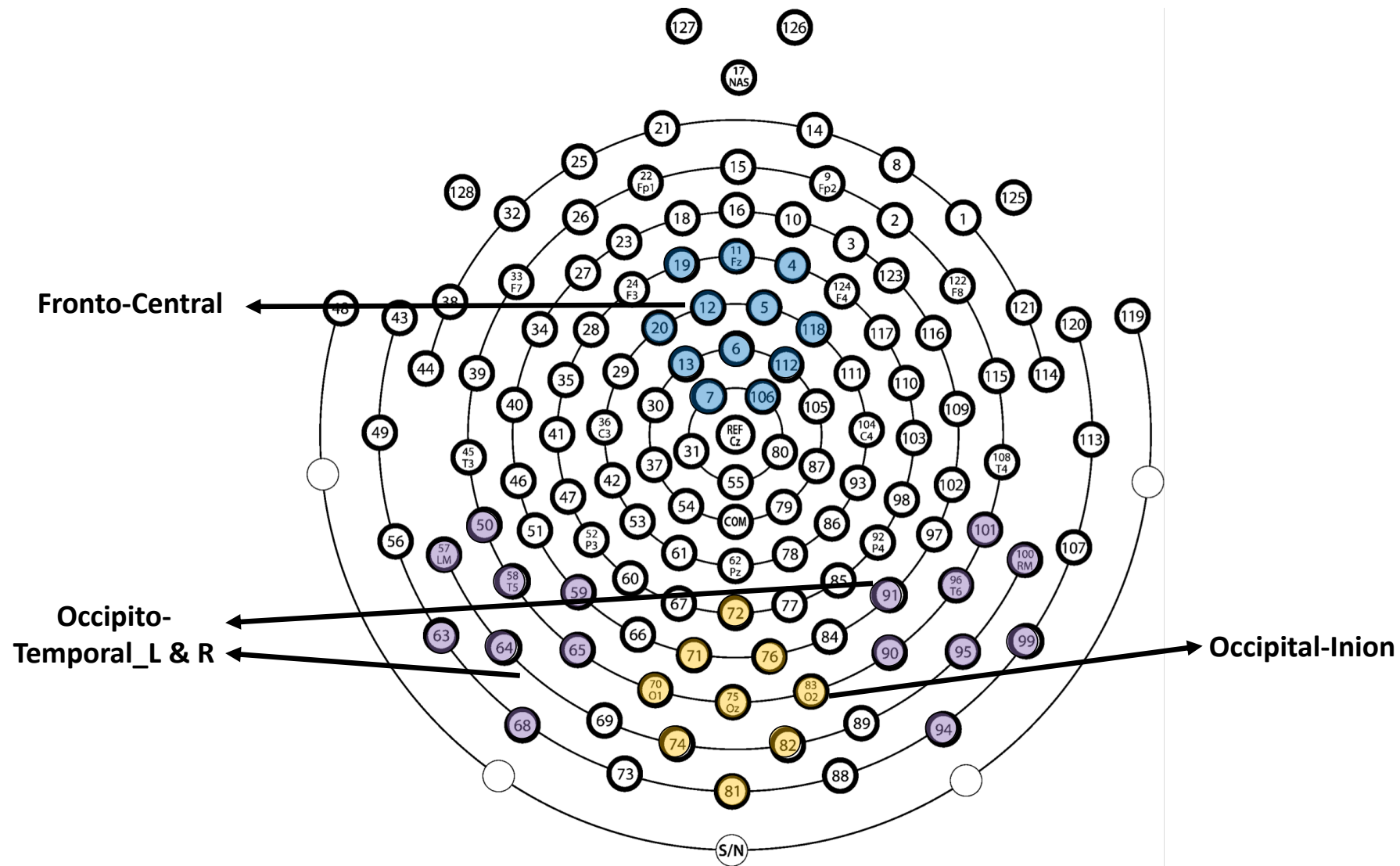

Supplement: Multimedia component 1 [file mmc1.pdf]
